# Systemic mesalazine treatment prevents spontaneous skin fibrosis in PLK2-deficient mice

**DOI:** 10.1007/s00210-021-02135-w

**Published:** 2021-08-19

**Authors:** Manja Newe, Theresa A. Kant, Maximilian Hoffmann, Johanna S. E. Rausch, Luise Winter, Karolina Künzel, Erik Klapproth, Claudia Günther, Stephan R. Künzel

**Affiliations:** 1 Institute of Pharmacology and Toxicology, Faculty of Medicine Carl Gustav Carus, Technische Universität Dresden, Fiedlerstraße 42, 01309 Dresden, Germany; 2 Department of Dermatology, Faculty of Medicine Carl Gustav Carus, Technische Universität Dresden, Dresden, Germany

**Keywords:** Fibrosis, Skin, Myofibroblasts, Collagen, Phenoconversion, Cytoskeleton

## Abstract

Skin fibrosis is a complex biological remodeling process occurring in disease like systemic sclerosis, morphea, or eosinophilic fasciitis. Since the knowledge about the underlying pathomechanisms is still incomplete, there is currently no therapy, which prevents or reverses skin fibrosis sufficiently. The present study investigates the role of polo-like kinase 2 (PLK2) and the pro-fibrotic cytokine osteopontin (OPN) in the pathogenesis of cutaneous fibrosis and demonstrates the antifibrotic effects of systemic mesalazine treatment *in vivo*. Isolated primary dermal fibroblasts of PLK2 wild-type (WT) and knockout (KO) mice were characterized *in* *vitro*. Skin thickness and histoarchitecture were studied in paraffin-embedded skin sections. The effects of mesalazine treatment were examined in isolated fibroblasts and PLK2 KO mice, which were fed 100 µg/g mesalazine for 6 months via the drinking water. Compared to WT, PLK2 KO fibroblasts displayed higher spontaneous myofibroblast differentiation, reduced proliferation rates, and overexpression of the fibrotic cytokine OPN. *In* *vitro*, 72 h of treatment with 10 mmol/L mesalazine induced phenotype conversion in PLK2 KO fibroblasts and attenuated OPN expression by inhibiting ERK1/2. *In vivo*, dermal myofibroblast differentiation, collagen accumulation, and skin thickening were prevented by mesalazine in PLK2 KO. Plasma creatinine levels indicated good tolerability of systemic long-term mesalazine treatment. The current study reveals a spontaneous fibrotic skin phenotype and ERK1/2-dependent OPN overexpression in PLK2 KO mice. We provide experimental evidence for the antifibrotic effectiveness of systemic mesalazine treatment to prevent fibrosis of the skin, suggesting further investigation in experimental and clinical settings.

**Supplementary Information:**

The online version contains supplementary material available at 10.1007/s00210-021-02135-w.

## Introduction

Skin fibrosis is the excessive deposition of extracellular matrix (ECM) proteins occurring either physiologically during wound healing or as a response towards pathological stimuli as found in localized (morphea) or systemic sclerosis, cutaneous graft-versus-host disease, eosinophilic fasciitis, and excessive scarring (keloid formation) (Gabrielli et al. 2009; Do and Eming 2016; Ihn 2019). A characteristic feature is excessive collagen accumulation leading to skin thickening and compromised function (Rockey et al. 2015). Affected patients suffer from physical as well as emotional pain due to disfiguration, contractures, and ulceration (Gabrielli et al. 2009; Sobolewski et al. 2019). The possible involvement of internal organs like the heart in systemic disorders such as systemic sclerosis, the lungs, or the kidneys contributes significantly to morbidity and mortality (Gabrielli et al. 2009; Sobolewski et al. 2019).

Fibroblasts are considered the major cellular regulators of tissue remodeling—independent of the site of injury (Baum and Duffy 2011; Rockey et al. 2015). Fibroblasts orchestrate connective tissue homeostasis and wound healing by secretion and degradation of collagens and other ECM proteins (Lynch and Watt 2018). Upon activation, resident tissue fibroblasts undergo a phenotypic transition towards myofibroblasts, which are characterized by increased secretory activity and the expression of fibrillary alpha-smooth muscle actin (αSMA) filaments (Baum and Duffy 2011; Poulet et al. 2016). Furthermore, transdifferentiation of adipocytes into myofibroblasts has been observed in dermal fibrosis and could account for the progressive replacement of the subcutaneous adipose tissue layer by connective tissue in systemic sclerosis (Marangoni and Lu 2017; Varga and Marangoni 2017). Remodeling of the cutaneous histo-architecture leads to increased tissue stiffness and due to increased diffusion distances to local hypoxia and subsequent epigenetic modifications, contributing to a self-sustaining cycle of myofibroblast activation and fibrosis (Distler et al. 2019).

Current pharmacological treatment for skin fibrosis includes the use of corticosteroids, methotrexate, UV irradiation, cyclophosphamide, mycophenolate mofetile, prostanoids, monoclonal antibodies, or, in severe cases of scleroderma, stem cell transplantation (Jordan et al. 2015; Del Papa et al. 2018; Sobolewski et al. 2019). The clinical outcome is often moderate with considerable side effects, leading to discontinuation of the therapy (Sobolewski et al. 2019; Panopoulos et al. 2020). Thus, the medical need for well-tolerable therapies that either prevent or reverse fibrosis is very high.

There is mounting evidence for shared fibrosis motifs and central regulatory pathways, which are relevant throughout the spectrum of fibroproliferative disease (Wernig et al. 2017; Distler et al. 2019). Thus, the “repurposing of targets,” which were found to be effective in one organ system, might be worthwhile in another as well. Central fibrotic pathways leading to fibroblast activation include but are not limited to TGF-β signaling and subsequent release of pro-fibrotic cytokines, the activation of nuclear receptors like NFƘB or PPARƔ, and initiation of the coagulation cascade (Distler et al. 2019; Hoffmann et al. 2020). The secreted ECM protein osteopontin (OPN), a downstream target of, i.e., TGF-β; ERK1/2 and NFƘB, is an emerging regulator of the fibrotic cascade (Kahles et al. 2014; Abdelaziz Mohamed et al. 2019). A detrimental role of elevated OPN levels has been demonstrated in cardiovascular, pulmonary and hepatic fibrosis (Ramadan et al. 2018; Abdelaziz Mohamed et al. 2019; Gui et al. 2020).

We previously found that loss of polo-like kinase 2 (PLK2), a serine-threonine kinase regulating cell cycle progression, cell survival, and metabolism (Ma et al. 2003; Matsumoto et al. 2009; Mochizuki et al. 2017) leads to ERK1/2-dependent overexpression of OPN and fibrotic remodeling of the heart and the lungs of PLK2-deficient mice (Kuenzel et al. 2020 (Abstract); Kant et al. 2021). Although there is growing experimental evidence that inhibition of OPN signaling yields pronounced antifibrotic effects (Wu et al. 2012; Ramadan et al. 2018), there is currently no clinically available drug targeting OPN specifically (Farrokhi et al. 2018).

We recently demonstrated antifibrotic effects of the aminosalicylate mesalazine by inhibiting ERK1/2 and thereby inducing phenotype conversion in cardiac myofibroblasts *in* *vitro* (Hoffmann et al. 2020). Additionally, a reduction of OPN gene expression after mesalazine treatment has been described in experimental liver fibrosis (Ramadan et al. 2018). Although its mode of action is not exclusively confined to a specific target, the favorable side effect profile and low cost of mesalazine prompt further investigation into antifibrotic drug repurposing (Hoffmann et al. 2020)*.*

Here, we characterize the cutaneous phenotype of PLK2 KO mice showing spontaneous fibrosis development and OPN overexpression as found in systemic sclerosis (Wu et al. 2012). Furthermore, we provide experimental evidence for the effectiveness and safety of systemic mesalazine treatment to prevent skin fibrosis *in vivo*.

## Materials and methods

### PLK2 wild-type and knockout mice

PLK2 WT and KO mice are commercially available via the Jackson Laboratory (129S.B6N-Plk2^tm1Elan^/J; The Jackson Laboratory, Bar Harbor, ME, USA). Animals were bred and kept according to the governmental and institutional animal welfare regulations (T 2014/5, TVA 25/2017, TVV 64/2018). To assess the effects of PLK2 KO on the skin, male and female homozygous PLK2 WT and KO mice were used in this study.

### Cell isolation

Primary dermal PLK2 WT and KO fibroblasts were isolated according to a previously published protocol (Künzel et al. 2019). For this purpose, skin samples of the ears of euthanized PLK2 WT and KO mice were used, as the ears display the least amount of fur and therefore the smallest risk of accidental contamination during isolation.

### Cell culture

All *in* *vitro* experiments were performed with isolated primary dermal fibroblasts from either PLK2 WT or PLK2 KO mice. Cells were cultured in non-coated cell culture dishes (Sigma Aldrich; USA; Techno Plastic Products, Switzerland) under controlled conditions (37 °C, 90% humidity, 5% CO_2_). Dulbecco’s modified Eagle medium (DMEM) with high glucose (4500 mg/L; Sigma Aldrich, USA) supplemented with 10% fetal calf serum (FCS) and 1% penicillin–streptomycin was used as control culture medium.

### *In vitro* mesalazine treatment protocol

**Fig. 1:**
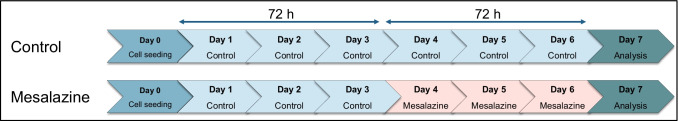
Schematic illustration of the *in* *vitro* mesalazine treatment protocol

After seeding, cells were cultured in control medium for 72 h, followed by either 72 h of medium (solvent control) or 10 mmol/L mesalazine (A3537, Sigma Aldrich, USA) solved in medium (Fig. 1). Medium and reagents were changed daily.

### Functional fibroblast characterization

#### Proliferation

To evaluate cell proliferation, 1*10^4^ cells/well were seeded in 12-well plates. Medium was changed daily as described above. After 5 and 10 days, cells were harvested using 0.25% trypsin and a Buerker counting chamber. Counting results are presented as cells*10^4^/mL.

#### Myofibroblast differentiation

Immunocytochemistry (ICC) for fibrillary αSMA was conducted as described previously (Poulet et al. 2016; Hoffmann et al. 2020; Kant et al. 2021) to quantify myofibroblast differentiation. Therefore, 1*10^4^ cells/well were seeded on glass coverslips in 24-well plates. Cell culture and mesalazine treatment were performed as described above. At the end of the experiment, cells were washed twice with cold PBS, fixated with 4% paraformaldehyde for 15 min, and washed again with cold PBS twice. After fixation, the following steps were performed (Table 1):

**Table. 1:** Fibroblast immunofluorescence staining protocol

Step	Description	Temperature
1	Permeabilization (15 min, 0.1% Triton-X)	RT^1^
2	Wash twice with PBS^2^	RT
3	Blocking (1 h, 10% FCS)	RT
4	Primary antibody (1 h in humidified chamber)	RT or 4 °C overnight
5	Wash twice with PBS	RT
6	Secondary antibody and DAPI^3^ (1 h in humidified chamber)	RT
7	Wash twice with PBS	RT
8	Mounting with 7–10 µL of Fluoromount G	RT
9	UV-protected storage until imaging	4 °C

^1^Room temperature. ^2^Phosphate-buffered saline. ^3^4′,6-Diamidin-2-phenylindol

The percentage of myofibroblasts was calculated in relation to the total number of counted nuclei from randomly selected areas of independent coverslips. Upon expression of fibrillary αSMA filaments, a cell was considered a myofibroblast (Poulet et al. 2016). At least 50 cells/coverslip were analyzed. Primary and secondary antibodies used in the present study are provided in Table 2.

**Table. 2:** Primary and secondary antibodies

Primary antibodies				
Protein	Dilution	Conjugate/source	Product-Nr	Usage
αSMA	1:200 (ICC)	Mouse	A5228	ICC^1^
ERK 1/2 (p42/44)	1:1000	Rabbit	#9102	WB^2^
Phospho-ERK 1/2 (p42/44)	1:1000	Rabbit	#9101	WB
Osteopontin	1:1000	Rabbit	ab8448	WB
EEF2	1:50.000	Rabbit	ab40812	WB
Secondary antibodies				
Goat-anti-rabbit	1:10.000	Peroxidase	111–035-045	WB
Alexa fluor 546 (Goat-anti-mouse)	1:400	Streptavidin	Z25004	ICC

^1^Immunocytochemistry. ^2^Western blot

### *In vivo* mesalazine long-term treatment

At 7 ± 2 weeks of age, male and female PLK2 WT and KO mice were randomly assigned to the treatment (100 µg/g mesalazine) or the solvent control group. All mice received water and food ad libitum. Mesalazine was dissolved in 20% hydrochloric acid (HCl), added to the drinking water and protected from UV exposure by tinfoil coating of the water bottles. Twenty percent HCl was used as solvent control. The body weight of all animals was measured weekly. To ensure constant dosage, the drinking amount was assessed three times a week. The dose of mesalazine was adjusted according to body weight and drinking amount. After 6 months of treatment, all mice were sacrificed and blood plasma and skin tissue samples were acquired. Selected effects of mesalazine treatment on PLK2 WT mice are displayed separately in the Supplement (see Supplementary Fig. 2).

### Histology, image acquisition, and analysis

Skin samples were fixated in 4% paraformaldehyde overnight for histological analysis. Subsequently, the samples were embedded in paraffin, sectioned (5-µm layer thickness), and stained (hematoxylin/eosin (H&E) and picrosirius red) by the staff of the histology facility at the Center for Molecular and Cellular Bioengineering (CMCB), Dresden. The immunohistochemical staining was performed as described previously (Kant et al. 2021). Fluorescence and brightfield images (for H&E and picrosirius red) were acquired with a Keyence BZ-X710 All-in-One Fluorescence Microscope (Keyence Corporation of America, USA) and a Zeiss Axio Observer Z1 microscope (Carl Zeiss AG, Germany). To quantify tissue collagen accumulation and tissue αSMA fluorescence intensity, FIJI 1.52n software (Schindelin et al. 2012) was used to quantify stained areas in relation to the total image area.

### Skin thickness measurement

To evaluate whole-skin thickness of PLK2 WT and KO mice, H&E-stained skin sections were measured at 3 randomly selected areas of the sample using the Bz X Analyzer Software (Keyence Corporation of America, USA). For each sample, mean values were calculated.

### SDS-PAGE, western blotting, and immunodetection

To extract protein from whole cell lysate, a radioimmunoprecipitation assay buffer (30 mM Tris, 0.5 mM EDTA, 150 mM NaCl, 1% NP-40, 0.1% SDS) supplemented with 10% protease and phosphatase inhibitors (Roche, Switzerland) was used. Protein concentration was measured using a bicinchoninic acid kit (Thermo Fisher, USA). To separate proteins via gel electrophoresis, 20 µg of whole cell protein was applied per lane of a 10% polyacrylamide gel and subsequently transferred to a nitrocellulose membrane. After incubation with primary and secondary antibodies (concentrations provided in Table 2), immunodetection was performed with a Fusion FX device (Vilber Lourmat Deutschland GmbH, Germany).

### Creatinine ELISA

A commercially available ELISA kit (ab65340, Abcam plc, UK) was performed according to the manufacturer’s protocol with EDTA plasma from PLK2 WT or PLK2 KO mice after long-term treatment with mesalazine.

### Units and data analysis

SI units were used throughout the manuscript. The measure “arbitrary units” (AU) was used in cases of unitless results. For data analysis and graphic representation, Prism 8 (GraphPad, USA) was used. Data are presented as single data points and mean ± standard error of the mean (SEM). For comparisons between two conditions, Student’s *t*-test was used with Welsh’s correction if appropriate. When comparing three or more conditions, a one-way ANOVA with Tukey posttest was performed. Two means were considered significantly different with *p*-values < 0.05. *, **, and *** indicate *p*-values below 0.05, 0.01, and 0.001, respectively.

## Results and discussion

### PLK2 KO leads to fibroblast activation and skin thickening

Previous research of our group focused on PLK2 as a regulator of tissue homeostasis and fibrotic remodeling in the cardiovascular system (Kuenzel et al. 2020 (Abstract); Kant et al. 2021). In the present study, we characterize the cutaneous phenotype of the PLK2 KO mouse model.

**Fig. 2:**
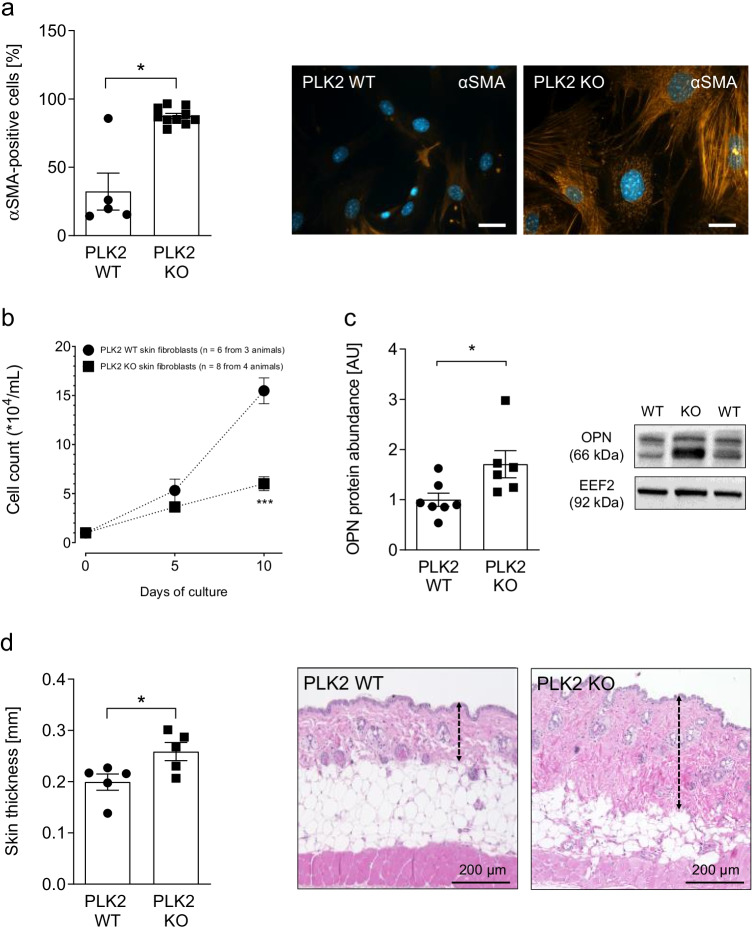
Genetic deletion of PLK2 induces a pro-fibrotic microenvironment in the skin. **a** Quantification and representative fluorescence images of myofibroblast differentiation in isolated primary dermal PLK2 WT and KO fibroblasts determined by immunocytochemistry for αSMA filaments (orange). The nuclei were stained blue with DAPI. The scale bars equal 20 µm. **b** Dermal fibroblast proliferation curves under basal conditions (*n* = 6 (WT) vs. 7 (KO)). **c** Representative western blot and quantification of osteopontin (OPN) protein expression in primary dermal PLK2 WT and KO fibroblasts under basal conditions (*n* = 7 (WT) vs. 6 (KO)). EEF2, eukaryotic elongation factor. **d** Quantification of skin thickness and representative H&E-stained histological skin sections of 4-month-old PLK2 WT and KO mice (*n* = 5 per group). The scale bars equal 200 µm

Myofibroblast differentiation is a key mechanism of fibrosis development (Baum and Duffy 2011; Distler et al. 2019). Myofibroblasts secrete ECM proteins, growth factors, and cytokines, leading to a self-sufficient cycle of fibroblast activation and tissue remodeling (Distler et al. 2019; Künzel et al. 2020). Isolated dermal PLK2 WT and KO fibroblasts were characterized with particular emphasis on myofibroblast differentiation and cell proliferation *in* *vitro*. Compared to WT, PLK2 KO fibroblasts displayed significantly higher spontaneous myofibroblast differentiation as determined by ICC for fibrillary αSMA (Fig. 2a, *p* < 0.05). Conversely, proliferation rates of PLK2 KO fibroblasts were significantly lower compared to WT at day 10 (Fig. 2b, *p* < 0.001).

The release of inflammatory cytokines presents a recurring and consistent motif in the development of fibrosis (Rockey et al. 2015; Distler et al. 2019). Thus, pathologically elevated OPN expression has been demonstrated to cause cardiac, pulmonary, hepatic, and dermal fibrosis (Pardo et al. 2005; Wu et al. 2012; Ramadan et al. 2018; Abdelaziz Mohamed et al. 2019). OPN protein expression was determined by western blot. In PLK2 KO fibroblast lysates, OPN expression was approximately twofold higher than in WT (Fig. 2c, *p* < 0.05) pointing at a pro-fibrotic cutaneous microenvironment (Wu et al. 2012).

To investigate, whether fibroblast activation induces early remodeling of the cutaneous histo-architecture in PLK2 KO, 1 × 1-cm skin biopsies were taken from the back area of euthanized 4-month-old PLK2 WT and KO mice. Histological analyses of H&E-stained skin demonstrated a significant increase in dermal thickness and a reduction of the subcutaneous adipose tissue layer in PLK2 KO compared to their WT littermates (Fig. 2d, *p* < 0.05). These findings reflect the pathophysiology of skin fibrosis in systemic sclerosis as fibrotic remodeling is initiated in the lower dermis and upper subcutaneous layer (Gabrielli et al. 2009) involving the transdifferentiation of adipocytes into myofibroblasts, leading to a progressive obliteration of subcutaneous fat and replacement by connective tissue (Fleischmajer et al. 1972; Varga and Marangoni 2017). At this point, it remains the subject of future research to clarify whether activated resident tissue fibroblasts invade the subcutaneous layer or adipocytes transdifferentiate into myofibroblasts. Previous research on PLK2 KO identified spontaneous pro-fibrotic remodeling of the lungs accompanied by an increase in ERK1/2 phosphorylation and OPN overexpression (Kant et al. 2021). Progression of interstitial lung disease is a major cause of death in patients suffering from systemic sclerosis (Volkmann and Fischer 2021). Although treatment with endothelin-receptor antagonists like bosenthan or vascular dilation with prostanoids ameliorates symptoms (Heresi and Minai 2008; Vlachou et al. 2021), a causal therapy is still absent. As remodeling of the skin, the heart, and the lungs occurs synchronously in PLK2 KO, this model could be leveraged in the future to study the mechanisms of systemic sclerosis progression and to identify putative treatments.

### Mesalazine induces fibroblast phenotype conversion *in vitro*

To date, clinically applicable therapies aiming at preventing or reversing fibrosis are widely absent (Hoffmann et al. 2020; Zhao et al. 2020). Experimental anti-OPN treatment to ameliorate fibrosis has delivered promising results in the heart, the liver, and the skin (Wu et al. 2012; Zhao et al. 2016; Ramadan et al. 2018). Genetic knockout of OPN has been shown to be effective at preventing experimentally induced scleroderma in mice (Wu et al. 2012). However, as a genetic approach is not feasible in patients, targeted anti-OPN treatment has been evaluated (Farrokhi et al. 2018). Due to relatively high OPN plasma concentrations and a short half-life in humans, high doses and short dosing intervals would be necessary, making antibody therapy currently not feasible (Farrokhi et al. 2018). Therefore, repurposing of established drugs might be a worthwhile approach to reduce OPN expression both safely and at acceptable costs in the near future (Paul et al. 2010; Sertkaya et al. 2016; Pushpakom et al. 2019).

**Fig. 3:**
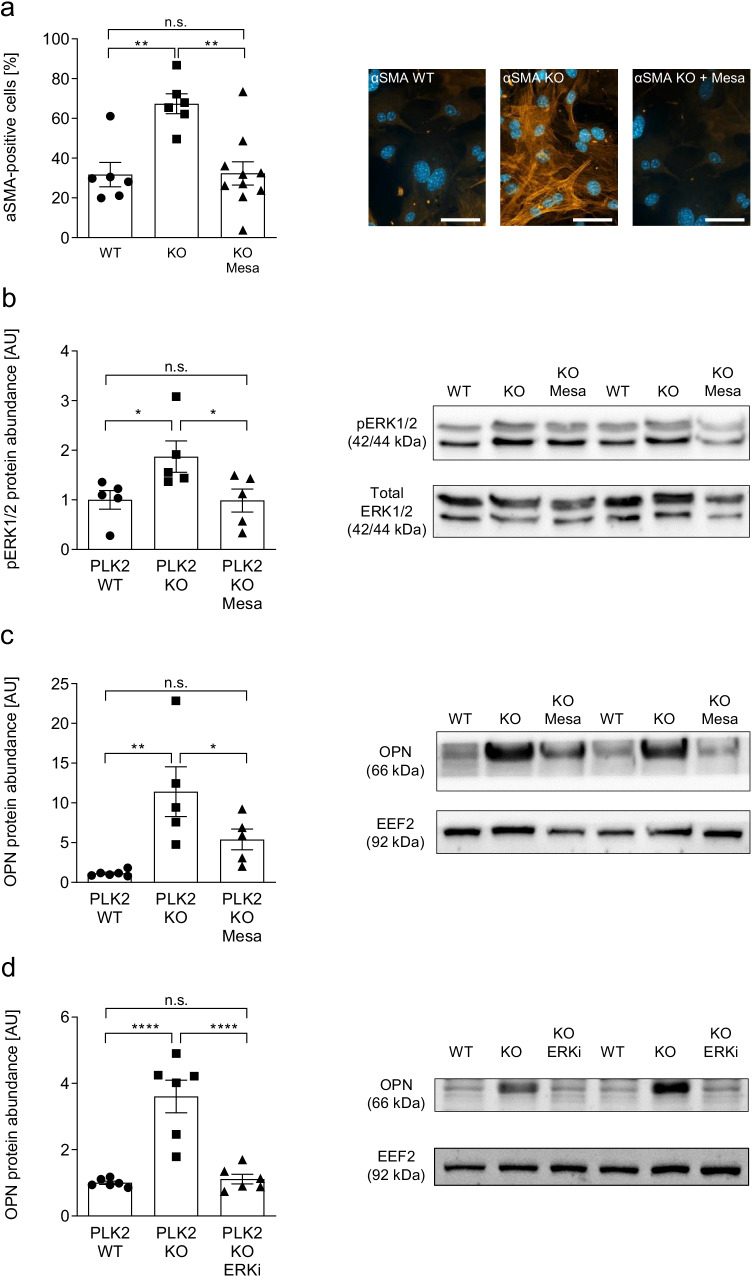
Mesalazine induces phenotype conversion *in* *vitro*. **a** Quantification and representative fluorescence images of myofibroblast differentiation in isolated primary dermal PLK2 WT and KO fibroblasts determined by immunocytochemistry for αSMA filaments (orange). The nuclei were stained blue with DAPI. All cells were cultured for 72 h under control conditions. Subsequently either mesalazine (10 mmol/L) or control (cell culture medium) treatment followed for additional 72 h (6 ≤ *n* ≤ 10 independent coverslips). The scale bars equal 20 µm. **b** Quantification and representative western blot of extracellular signal-regulated kinases1/2 (ERK1/2) phosphorylation in primary dermal PLK2 WT and KO fibroblasts under basal conditions and upon 72-h mesalazine treatment (10 mmol/L) (*n* = 5 per group). **c** Quantification and representative western blot of OPN protein expression in primary dermal PLK2 WT and KO fibroblasts under basal conditions and upon 72-h mesalazine treatment (10 mmol/L) (*n* = 5 per group). d Quantification and representative western blot of OPN protein expression in primary dermal PLK2 WT and KO fibroblasts under basal conditions and upon treatment with the specific ERK1/2 inhibitor SCH772984 at 10 nmol/L for 72 h (*n* = 6 per group)

We previously demonstrated that *in* *vitro* mesalazine treatment is sufficient to induce phenotype conversion in TGF-β-stimulated cardiac myofibroblasts (Hoffmann et al. 2020). Therefore, we tested the effects of pharmacological treatment with 10 mmol/L mesalazine in PLK2 KO fibroblasts on differentiation. Consistent with the results presented in Fig. 2a, PLK2 KO fibroblasts displayed significantly increased myofibroblast differentiation (Fig. 3a, *p* < 0.01). After 72 h of mesalazine treatment, fibrillary αSMA expression was markedly reduced in PLK2 KO and not significantly different from WT anymore (Fig. 3a, *p* < 0.01).

Activation of ERK1/2 is a central step in the fibrotic cascade, initiating myofibroblast differentiation and OPN expression (Kahles et al. 2014; Distler et al. 2019). The family of polo-like kinases negatively regulates ERK1/2 activity under physiological conditions by inhibiting the Ras pathway upstream of ERK1/2 (Lee et al. 2011; Li et al. 2012). Accordingly, we found significantly higher ERK1/2 phosphorylation in PLK2 KO compared to WT under basal conditions (Fig. 3b, *p* < 0.05). This finding is in line with published data on ERK1/2 activation in PLK2-deficient primary pulmonary fibroblasts (Kant et al. 2021).

We previously demonstrated that mesalazine acts as a multi-target inhibitor in central fibrotic pathways, i.e., ERK1/2, NFƘB, or SMAD2/3 (Hoffmann et al. 2020). Confirming the results of our previous study on cardiac fibroblasts, 72 h of mesalazine treatment significantly reduced ERK1/2 phosphorylation (Fig. 3b, *p* < 0.05). In line, OPN protein expression was similarly reduced by mesalazine treatment (Fig. 3c, *p* < 0.05). To mechanistically prove that OPN protein expression in dermal PLK2 KO fibroblasts is ERK1/2-dependent, we treated dermal PLK2 KO fibroblasts with the selective ERK1/2 inhibitor SCH772984 at 10 nmol/L for 72 h *in* *vitro*. Compared to PLK2 KO fibroblasts which were treated with solvent control, OPN protein expression was reduced to WT levels by selective pharmacological ERK1/2 inhibition (Fig. 3d, *p* < . 0.0001). This finding is consistent with previous reports that ERK1/2 activity is crucial for OPN expression (Beck and Knecht 2003; Kahles et al. 2014). However, regulation of OPN transcription is a multifactorial process (Kahles et al. 2014). Therefore, at this point, we cannot rule out that inhibition of other regulators than ERK1/2 by mesalazine may as well contribute to the observed reduction of OPN expression.

### Systemic mesalazine treatment prevents fibrotic remodeling of the skin

**Fig. 4:**
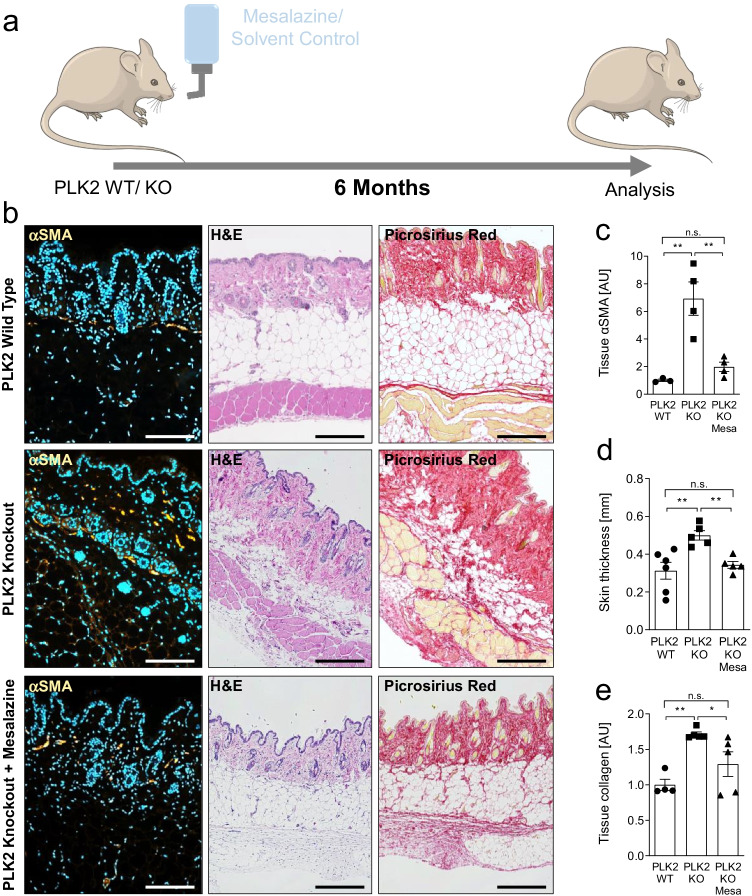
Oral long-term mesalazine treatment prevents skin fibrosis in PLK2 KO mice. **a** Schematic illustration of the treatment regimen (modified from Servier Medical Art, licensed under a Creative Commons Attribution 3.0 Unported License. http://smart.servier.com/). **b** Representative images of paraffin-embedded skin tissue sections. The scale bars equal 200 µm. Left panel: Representativeimmunofluorescence images for αSMA (orange). The nuclei were stained blue with DAPI. Mid panel: Representative H&E stainings. Right panel: Representative picrosirius red stainings for collagen. **c** Quantification of tissue αSMA. **d** Quantification of skin thickness. **e** Quantification of tissue collagen

Next, we tested whether mesalazine treatment is sufficient to prevent skin fibrosis development in PLK2 KO mice. For the treatment of chronic inflammatory bowel disease, mesalazine is either administered rectally or as enteric-coated formulation to avoid systemic resorption and to guarantee high local bioavailability in the colon (Ye and van Langenberg 2015). A topical formulation for mesalazine-based therapy of skin conditions would be desirable to reduce potential systemic side effects (Chowdhury 2013). However, as there is no clinically available topical mesalazine formulation, we chose to administer the drug systemically via the drinking water for a period of 6 months. PLK2 KO mice were randomly assigned to the treatment (100 µg/g mesalazine) or the solvent control group. WT mice received solvent control only. After 6 months of continuous treatment, tissue biopsies and blood samples were taken (Fig. 4a).

First, we investigated the impact of mesalazine on tissue myofibroblast differentiation via ICC for fibrillary αSMA on paraffin-embedded skin sections. Compared to WT, the fluorescence signal for αSMA was significantly stronger in PLK2 KO (Fig. 4b left panel and c, *p* < 0.01). In mesalazine-treated skin sections, the αSMA signal intensity was not significantly different from WT (Fig. 4b left panel and c), suggesting that aberrant myofibroblast differentiation was absent, confirming our previous *in* *vitro* results in cardiac fibroblasts (Hoffmann et al. 2020).

The histological analysis of H&E-stained paraffin-embedded skin biopsies indicated a significantly higher skin thickness in PLK2 KO compared to WT and an age-dependent thickening of the skin in both genotypes (KO: 0.49 ± 0.03 mm, WT: 0.31 ± 0.04 mm; Fig. 4b mid panel and c, *p* < 0.01). Conversely, skin thickness of mesalazine-treated PLK2 KO mice was not significantly different from WT (KO + mesalazine: 0.34 ± 0.01 mm, WT: 0.31 ± 0.04 mm; Fig. 4b and c, *p* > 0.05).

Collagen deposition was determined by picrosirius red staining. Compared to their WT littermates, PLK2 KO mice displayed significantly higher and irregular collagen accumulation in the dermis and hypodermis (Fig. 4b right panel and e, *p* < 0.01) consistent with histological data from scleroderma patients (Gabrielli et al. 2009; Sobolewski et al. 2019). Both irregular collagen deposition and remodeling of the subcutaneous adipose tissue layer were absent in mesalazine-treated animals. This finding in particular could be relevant for patients, as the progressive reduction of the subcutaneous fat accelerates skin stiffening and loss of mobility and function (Thaller et al. 1990; Xiong et al. 2017) and current studies evaluate the therapeutic potential of preventing adipocyte-to-myofibroblast differentiation in the skin (Mastrogiannaki et al. 2016; Varga and Marangoni 2017).

Although the skin of PLK2 KO mice displays a distinct fibrotic substrate, fibrosis severity appears to be less pronounced than in human scleroderma. Our data suggests an early genetic predisposition towards skin fibrosis induced by loss of PLK2 with age-dependent progression (Figs. 2 and 4). This feature could be considered an advantage of the model, as many established mouse models of skin fibrosis rely on experimental induction (Do and Eming 2016). To study a maximal phenotype, however, a “multiple-hit model” (B Moore et al. 2013; Kant et al. 2021) with, e.g., dermal bleomycin or hypochloric acid injections (Marangoni et al. 2016) could help to answer mechanistic questions and to study potential drug targets and treatment options in PLK2 KO mice already at an earlier age.

### Safety data for systemic long-term mesalazine treatment

**Fig. 5:**
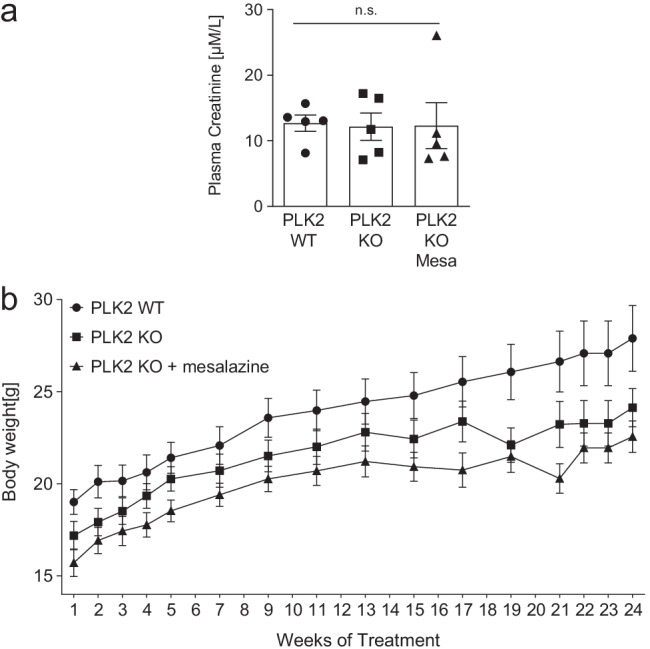
Safety parameters of systemic long-term mesalazine treatment. **a** Quantification of plasma creatinine levels in PLK2 WT and KO mice upon solvent control or mesalazine (100 µg/g body weight) treatment determined by ELISA from EDTA plasma. **b** Body weight development of PLK2 and WT mice upon solvent control or mesalazine (100 µg/g body weight) treatment

In chronic inflammatory bowel disease, mesalazine induces topical therapeutic effects at the intestinal mucosa. Thus, systemic side effects can be reduced (Ye and van Langenberg 2015). As we administered mesalazine systemically, we put particular emphasis on putative adverse events in animals. Aminosalicylates, such as mesalazine, have been reported to be potentially nephrotoxic (Gisbert et al. 2007). Therefore, we measured plasma creatinine levels at the end of the 6-month treatment period with ELISA. Creatinine levels were not significantly different between PLK2 WT, KO, and mesalazine-treated KO (Fig. 5a). Furthermore, gastrointestinal side effects like diarrhea, reduced appetite, or nausea can occur during mesalazine treatment (Turunen et al. 1987; Brunton et al. 2018). Thus, all animals were weighed weekly throughout the experiment. Although the body weight of PLK2 KO mice was lower than the body weight of the respective WT littermates, all groups displayed progressive weight development throughout the experiment (Fig. 5b). Lastly, no indicators (loss of hair, neglect of fur care, or mutilation) of otherwise harmful side effects were visible during the experiment. Taken together, our results confirm that the use of non-enteric-coated mesalazine against skin fibrosis is well-tolerable in mice.

### Potential limitations

Although downregulation of PLK2 has been described in human cardiac fibrosis and different entities of human pulmonary fibrosis (Kuenzel et al. 2020 (Abstract); Kant et al. 2021), it remains to be investigated whether PLK2 expression is downregulated in fibroproliferative skin disease such as systemic sclerosis. Here, we focused on the reduction of OPN expression by mesalazine as targeting OPN is a currently pursued approach in fibrosis research, which has not been achieved, yet (Farrokhi et al. 2018). We are aware that other cytokines and cellular mediators may as well contribute to the fibrotic skin phenotype in PLK2 KO. As presented in Supplementary Fig. 1, there is also an increase in *TGF-β* and *TNFα* gene expression in PLK2 KO. At the current stage, however, we cannot draw mechanistic conclusions on their impact on the observed phenotype. These mediators and pathways remain the subject of future research on this topic. Based on our previous findings (Hoffmann et al. 2020), we studied the effects of the aminosalicylate mesalazine on skin fibrosis development in the present study. Conceivably, other salicylates such as acetylsalicylic acid (ASS) could have similar anti-fibrotic effects (Liu et al. 2017). However, potentially severe adverse effects might limit their use. As mesalazine is considered the effective moiety of sulfasalazine (Klotz et al. 1980) and ASS would require high doses in humans (Liu et al. 2017), increasing the likelihood of serious adverse events like bleeding, we are convinced that the use of mesalazine is reasonable in future translational research.

### Conclusion and clinical implications

In the present study, we provide experimental evidence that oral mesalazine treatment is effective to prevent cutaneous fibrosis development in genetically predisposed mice. We demonstrate that loss of PLK2 function induces spontaneous fibrotic remodeling of the skin due to aberrant myofibroblast activation and collagen accumulation. Although it remains to be investigated, whether loss of PLK2 function is present in human skin fibrosis, the PLK2 KO mouse model presents characteristic pathological features such as progressive skin thickening, obliteration of the subcutaneous adipose tissue and overexpression of OPN, which has emerged as a relevant mediator of fibrotic remodeling in various disease entities (Wu et al. 2012; Ramadan et al. 2018; Abdelaziz Mohamed et al. 2019). *In* *vitro*, mesalazine treatment led to phenotype conversion of myofibroblasts and a marked reduction of OPN expression by inhibiting ERK1/2 phosphorylation, confirming recent data (Hoffmann et al. 2020). *In vivo*, tissue myofibroblast differentiation, collagen accumulation, and subsequent skin thickening were prevented by 6 months of systemic mesalazine treatment. Kidney function as determined by plasma creatinine was sustained during mesalazine treatment and progressive gain of weight in treated animals indicated gastrointestinal tolerability. Mesalazine has been used in the clinical routine for decades and is generally well-tolerated and cost-effective (Hoffmann et al. 2020). Thus, mesalazine is an excellent candidate for drug repurposing in clinical trials and could be the basis for the development of new therapies against skin fibrosis.

## Supplementary information

Below is the link to the electronic supplementary material.

Supplementary file1 (PZFX 106 KB)

Supplementary file2 (PZF 657 KB)

Supplementary file3 (PZF 235 KB)

## Data Availability

All data analyzed during this study are included in this article as single values within the graphs.
